# Pch2 Acts through Xrs2 and Tel1/ATM to Modulate Interhomolog Bias and Checkpoint Function during Meiosis

**DOI:** 10.1371/journal.pgen.1002351

**Published:** 2011-11-03

**Authors:** Hsuan-Chung Ho, Sean M. Burgess

**Affiliations:** Department of Molecular and Cellular Biology, University of California Davis, Davis, California, United States of America; Waksman Institute, United States of America

## Abstract

Proper segregation of chromosomes during meiosis requires the formation and repair of double-strand breaks (DSBs) to form crossovers. Repair is biased toward using the homolog as a substrate rather than the sister chromatid. Pch2 is a conserved member of the AAA^+^-ATPase family of proteins and is implicated in a wide range of meiosis-specific processes including the recombination checkpoint, maturation of the chromosome axis, crossover control, and synapsis. We demonstrate a role for Pch2 in promoting and regulating interhomolog bias and the meiotic recombination checkpoint in response to unprocessed DSBs through the activation of axial proteins Hop1 and Mek1 in budding yeast. We show that Pch2 physically interacts with the putative BRCT repeats in the N-terminal region of Xrs2, a member of the MRX complex that acts at sites of unprocessed DSBs. Pch2, Xrs2, and the ATM ortholog Tel1 function in the same pathway leading to the phosphorylation of Hop1, independent of Rad17 and the ATR ortholog Mec1, which respond to the presence of single-stranded DNA. An N-terminal deletion of Xrs2 recapitulates the *pch2Δ* phenotypes for signaling unresected breaks. We propose that interaction with Xrs2 may enable Pch2 to remodel chromosome structure adjacent to the site of a DSB and thereby promote accessibility of Hop1 to the Tel1 kinase. In addition, Xrs2, like Pch2, is required for checkpoint-mediated delay conferred by the failure to synapse chromosomes.

## Author Summary

Sexually reproductive organisms utilize meiosis to produce gametes (e.g. egg and sperm). During meiosis, chromosome numbers reduce to half (haploid) and fertilization restores their numbers to a diploid state so that ploidy can be maintained throughout generations. Meiosis involves two successive divisions (meiosis I and meiosis II) that follow a single round of DNA replication. In meiosis I homologous chromosomes segregate, whereas in meiosis II sister chromatids segregate. Failure to properly segregate chromosomes leads to the formation of aneuploid gametes, which are a leading cause of birth defects and pregnancy loss in humans. In most organisms, proper chromosome segregation in meiosis I requires meiotic recombination, where the repair of deliberately introduced double-strand breaks (DSBs) generates physical connections between homologous chromosomes. Importantly, DSBs must be repaired in a timely fashion and coordinated with the meiotic cycle by the recombination checkpoint. Here we investigated the role of Pch2, an AAA^+^-ATPase protein, in regulating chromosome events during meiotic prophase. We found Pch2 functions with Tel1 (homolog of ATM) and the MRX component Xrs2 to signal blunt-ended, unprocessed DSB intermediates of meiotic recombination. In addition, physical interaction between Pch2 and Xrs2 appears to play additional roles during meiosis, independent of Tel1 function.

## Introduction

Meiosis is a specialized cell division program to produce haploid gametes. To achieve faithful chromosome segregation during meiosis I (MI), cells utilize meiotic recombination to establish physical connections through the formation of chiasmata or crossing-over at the DNA level between homologous chromosomes [1].

In budding yeast, meiotic recombination is initiated by programmed double-strand breaks (DSBs) catalyzed by a topoisomerase II-like enzyme Spo11 [2]. The 5′ ends of DSBs are resected to produce 3′ single-stranded DNA, at which Dmc1 and Rad51 load to mediate strand exchange with a homologous DNA sequence [3], [4]. Unlike in vegetative cells where sister chromatids are preferred templates for DSB repair, most meiotic programmed DSBs are repaired using homologous non-sister chromatids [5], [6], [7]. A subset of DSBs is repaired to form crossovers (CO) through a double Holliday junction (dHJ) pathway [8], [9], [10]. CO formation and distribution is highly regulated during meiosis; each homolog must receive at least one CO to sustain reductional segregation in meiosis I [11].

Interhomolog bias is established and maintained by regulatory proteins associated with chromosome axis structures, including Hop1 and Mek1. In response to DSBs, the meiotic chromosome axis protein Hop1 is phosphorylated by Tel1/Mec1 (ATM/ATR homologs) [12]. Phosphorylated Hop1 promotes dimerization and auto-activation of Mek1 kinase [13], [14], [15], [16]. A Hop1 mutant that is refractory to Tel1/Mec1 phosphorylation fails to activate Hop1-dependent Mek1 phosphorylation and results in the loss of interhomolog bias [12]. Mek1 kinase plays dual roles by promoting interhomolog bias and checkpoint signaling in the presence of recombination intermediates [13].

The presence of unrepaired DSBs is monitored by DNA damage checkpoint proteins Mec1, Rad17, Rad24, Tel1, and the MRX (Mre11-Rad50-Xrs2) complex [17]. Mutants defective in the repair of meiosis-induced DSBs activate one or more pathways involving these proteins [17]. Different lesions appear to activate different checkpoint pathways. For example, unresected DSBs appear to activate a checkpoint requiring Tel1 (ATM homolog) while unrepaired resected breaks activate a Mec1 (ATR) pathway [18], [19].

Pch2 is a member of the AAA^+^-ATPase family of proteins and is implicated in a number of meiosis-specific processes in budding yeast *Saccharomyces cerevisiae*, including meiotic recombination, chromosome axis formation, checkpoint signaling, crossover control and interhomolog bias [20], [21], [22], [23], [24]. Pch2 participates in one branch of a bifurcated pathway that defines the recombination checkpoint: One branch is regulated by Rad17 and Mec1, likely in response to ssDNA [19]. A second branch is regulated by Pch2, however, the activating lesion has not been defined [20]. In mouse the Pch2 homolog TRIP13 plays roles in axis morphogenesis and early steps of recombination [25], [26], [27]. In *Caenorhabditis elegans* and *Drosophila melanogaster,* PCH-2 plays a role in a checkpoint that monitors synapsis and/or axis formation [28], [29], [30]. Whether these seemingly disparate roles of Pch2 share mechanisms in common is an open question.

Pch2 was originally identified by mutation as a suppressor of the arrest/delay phenotype conferred by the deletion of *ZIP1* [31], which encodes the transverse element of the synaptonemal complex (SC) [32], [33]. Suppression of the *zip1*Δ delay phenotype by *pch2*Δ is enigmatic since the *zip1*Δ delay is also suppressed by deletion of *RAD17* [20]. Multiple roles for Zip1 during meiosis are indicated by the pleiotropic phenotypes associated with the deletion mutation [1], [34], therefore it is possible that Pch2 might signal more than one lesion during a challenged meiosis.

Our data support these key findings: 1. Pch2 and Rad17 contribute to suppression of intersister recombination through independent pathways with partially overlapping functions. 2. Pch2 and Tel1 function in the same epistasis pathway to regulate meiotic recombination checkpoint signaling, independent of Rad17 and Mec1. 3. Pch2 functions to signal the presence of unresected breaks leading to the phosphorylation of Hop1. 4. Pch2 physically interacts with the N-terminal region of Xrs2 containing putative BRCT repeats. Deletion of this non-essential region of Xrs2 leads to a defect in Pch2-dependent checkpoint signaling. 5. Xrs2 and Pch2 play a role in the synapsis checkpoint while Tel1 does not. These findings link multiple roles of Pch2 in budding yeast to the ATM homolog Tel1 and/or the MRX component Xrs2. We propose that phosphorylation of the meiotic chromosome axis protein Hop1 depends on two partially redundant pathways: one pathway involving Tel1, Pch2 and Xrs2 that responds to the presence of unprocessed DSBs and another pathway involving Mec1 and Rad17 that responds to the presence of resected DSB intermediates of homologous recombination.

## Results

### Pch2 and Rad17 prevent intersister repair during meiotic recombination

**Figure 1:**
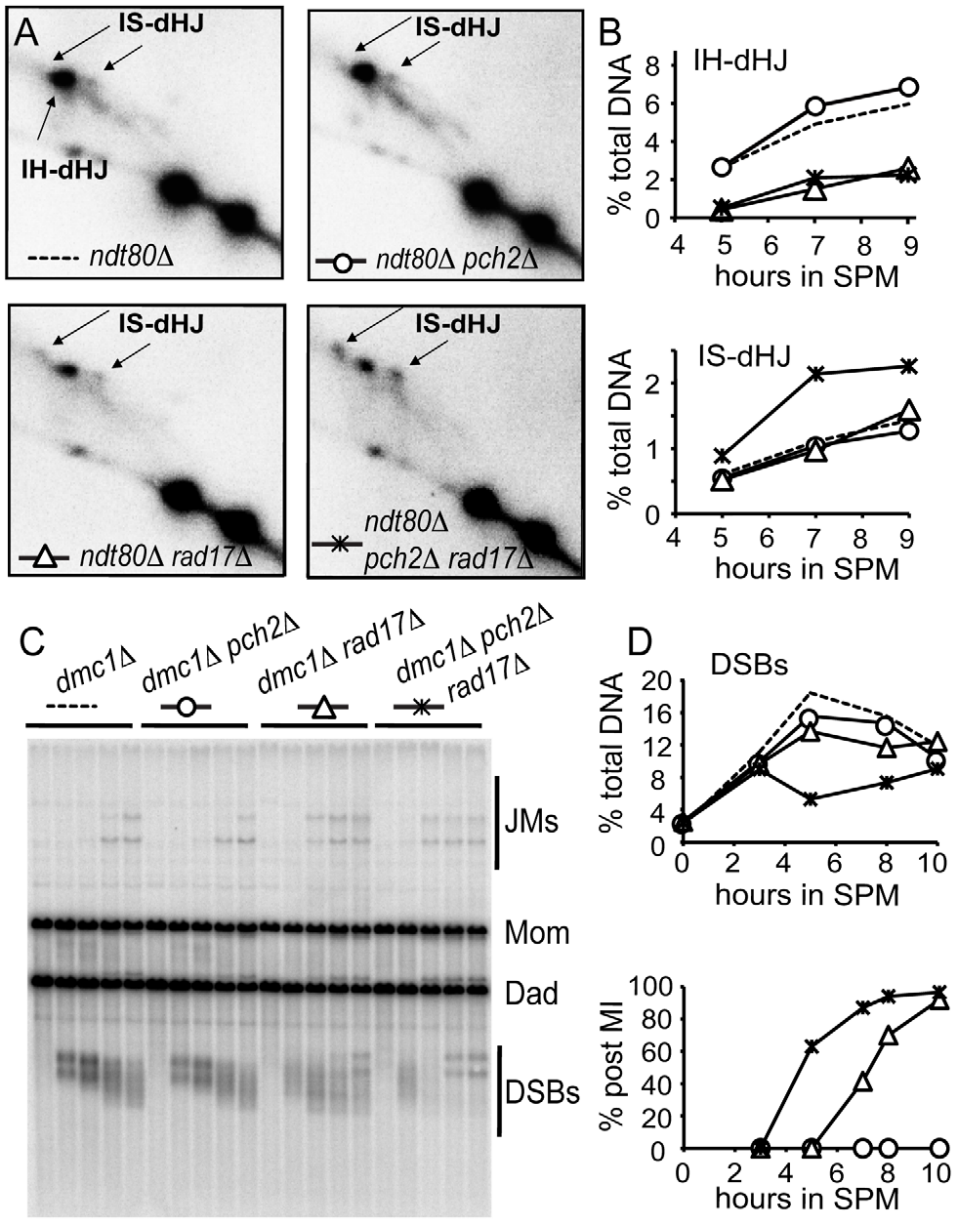
Pch2 and Rad17 prevent intersister repair. (A) Southern blot of 2D gel analysis of joint molecules in indicated strains 9 hr after transfer to SPM. (B) Quantitation of interhomolog double Holliday junctions (IH-dHJs) and intersister double Holliday junctions (IS-dHJs) as a percent of total DNA isolated from synchronized meiotic cultures at the indicated times after transfer to SPM (A). (C) Southern blot of 1D gel analysis of DSB turnover in indicated strains. The slow-migrating DSB species at late time points in *dmc1Δ rad17Δ* and *dmc1Δ pch2Δ rad17Δ* are likely DNA hairpin structures [35]. Rad17 may be involved in limiting formation of these structures. (D) Quantitation of DSBs (% total DNA) and percentage of cells that have completed at least the first meiotic division (post-MI) from the time course shown in (C).

Deletion of both *PCH2* and *RAD17* causes a synergistic reduction in spore viability and accelerated meiotic progression compared to either single mutant or wild type. Spore inviability is suppressed in a *spo13* mutant background suggesting that programmed DSBs are repaired, most likely using the sister chromatid as a template [20]. These combined phenotypes led us to suggest that Pch2 and Rad17 function in redundant pathways to suppress the use of sister chromatids to repair meiotic programmed DSBs. To test this, we monitored the presence of intersister (IS) and interhomolog (IH) joint molecules that form as intermediates of meiotic DSB repair at the *HIS4LEU2* hot spot in *pch2*Δ, *rad17*Δ and *pch2Δ rad17*Δ at various time points during meiotic progression in a synchronized cell culture using two-dimensional gel electrophoresis (Figure 1A, 1B). To detect maximal levels of these intermediates we used an *ndt80*Δ mutant background to block the resolution of dHJs to crossover products [8], [35]. While the *ndt80Δ pch2*Δ mutant gave ∼10% higher levels of IH-dHJ compared to the *ndt80*Δ strain, the levels in *ndt80Δ rad17*Δ and *ndt80Δ pch2Δ rad17*Δ were reduced by ∼60% and 67%, respectively. By contrast, while the *ndt80Δ pch2*Δ mutant gave ∼9% lower levels of IS-dHJ compared to the *ndt80*Δ strain, this species was increased in *ndt80Δ rad17*Δ and *ndt80Δ pch2Δ rad17*Δ mutants by ∼13% and 52%, respectively (based on averages of measurements from two independent time course experiments). Together these results suggest that Pch2 and Rad17 have independent and partially overlapping functions in promoting interhomolog bias.

In an independent test, we measured DSB levels in the *dmc1*Δ mutant background where DSBs form and are resected but their repair is blocked [3]. If the process of upholding interhomolog bias is compromised then breaks can be repaired using sister chromatids [7]. We found that steady-state DSB levels were decreased over two-fold in *dmc1Δ pch2Δ rad17*Δ compared to *dmc1Δ pch2*Δ and *dmc1Δ rad17*Δ (5 hours after transfer to SPM; Figure 1C and 1D). The observed decrease in DSBs in *dmc1Δ pch2Δ rad17*Δ (compare t = 3 hours and t = 5 hours), but not in *sae2Δ pch2Δ rad17*Δ where DSBs are not processed [20], suggests that repair of DSBs occurs using a sister chromatid. These results suggest that Pch2 and Rad17 are required to uphold the barrier to sister chromatid recombination.

### Pch2 and Rad17 promote Hop1 phosphorylation and Mek1 activation

**Figure 2:**
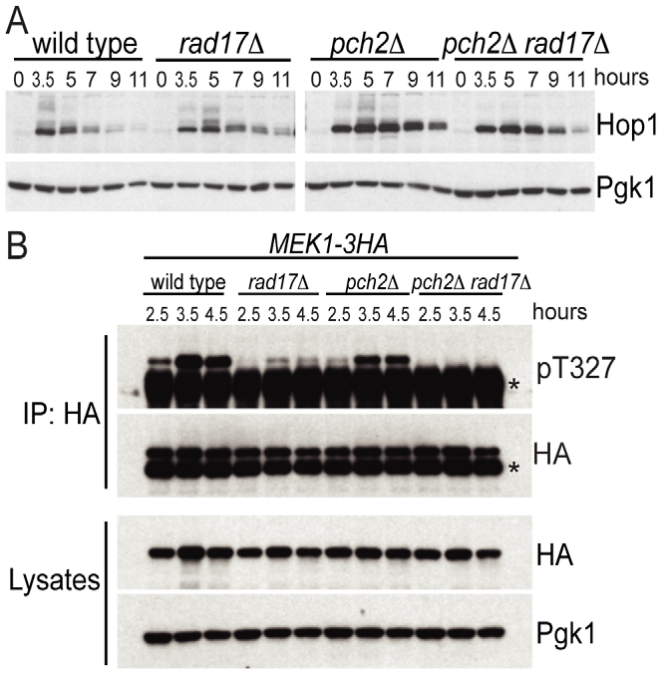
Pch2 and Rad17 promote Hop1 and Mek1 activation. (A) Western blot analysis of WT, *rad17Δ*, *pch2Δ* and *pch2Δ rad17Δ* at indicated time points after transfer to SPM using α-Hop1 antibody. Pgk1 Western blot was used as the loading control. The phosphorylated isoforms of Hop1 are detectable as slow-moving species. (B) Mek1–3HA immunoprecipitates from WT, *rad17Δ*, *pch2Δ* and *pch2Δ rad17Δ* at indicated time points were analyzed by Western blot using α-phospho-Akt substrate (recognizing pT327 of Mek1) and α-HA antibodies. *IgG heavy chain. Cell lysates were analyzed by Western blot using α-HA and α-Pgk1 antibodies.

From the findings above, we reasoned that Pch2 and Rad17 might independently promote phosphorylation of Hop1 in response to DSBs. In wild-type cells, Hop1 was transiently phosphorylated starting at about t = 3.5, as revealed by slow-migrating bands in a western blot using an α-Hop1 antibody (Figure 2A). Slow-moving Hop1 isoforms were abundant in both *pch2*Δ and *rad17*Δ single mutants but dramatically reduced in the *pch2Δ rad17*Δ double mutant. These results suggest that Pch2 and Rad17 function in different pathways leading to Hop1 phosphorylation.

We next examined the phosphorylation status of Mek1 using an α-Akt-substrate antibody to the T327 residue in the activation loop [36]. While phosphorylation of the T327 residue was present in the *pch2*Δ and *rad17*Δ single mutants, it was completely abolished in the *pch2Δ rad17*Δ double mutant, similar to the results seen above for Hop1 (Figure 2B). The reduction in Mek1–3HA phosphorylation in *rad17*Δ was more dramatic than the reduction of Hop1 phosphorylation in the same background. One interpretation of this result is that Rad17 not only regulates signaling upstream of Hop1 but also impacts the Hop1-dependent autophosphorylation of Mek1. Consistent with this notion, *rad17*Δ shows aberrant SC formation [37] perhaps indicating aberrantly formed axial elements. Together, these results demonstrate that two independent pathways defined by Pch2 and Rad17, respectively, regulate the activation status of the meiotic chromosome axis proteins Hop1 and Mek1. The failure to phosphorylate Hop1 and Mek1 in the absence of both Pch2 and Rad17 may account for the loss of interhomolog bias in the *pch2Δ rad17*Δ double mutant background.

### Pch2 acts together with Tel1 to promote spore viability and normal MI division timing

A hallmark of mutants defective in interhomolog bias is the formation of largely inviable spore products due to reduced levels of interhomolog crossovers [1]. Consistent with this pattern, the *pch2Δ rad17*Δ double mutant gives <0.1% viable spores, while each single mutant gives higher levels (37.1% for *rad17*Δ and 92.2% for *pch2*Δ; Table 1) [20]. Like Rad17 and Pch2, the ATR/ATM homologs Mec1 and Tel1 have also been shown to play partially redundant roles in meiotic interhomolog recombination by phosphorylating Hop1 [12]. Since *RAD17* and *MEC1* are in the same epistasis group that mediates checkpoint signaling in the presence of ssDNA [19], [37], one possibility is that Pch2 functions with Tel1 in a separate pathway, perhaps in response to unresected DSBs [18]. To test this, we examined spore viability in mutants containing pair-wise combinations of *pch2*Δ, *rad17*Δ, *tel1*Δ and *mec1*Δ mutations. In the cases where we predicted the two genes would act in the same pathway (e.g. *pch2Δ tel1*Δ and *rad17Δ mec1Δ)*, there was no decrease in spore viability compared to the single mutants (Table 1). By contrast, in the cases where we predicted the two genes would function in different pathways we observed a synergistic decrease in spore viability in the double mutants (2.9% for *pch2Δ mec1*Δ and <0.1% for *rad17Δ tel1*Δ).

**Table 1:** Spore viability.

StrainSBY#^1^	Genotype	% sporeviability^3^	# of spores
1903	WT	96.9	128
2351	*pch2Δ*	92.2	128
2368	*rad17Δ*	37.1	140
3798	*tel1Δ*	91.0	212
3815	*mec1Δ*	70.3	212
2361	*pch2Δ rad17Δ*	<0.1	140
3801	*pch2Δ tel1Δ*	92.9	212
3822	*pch2Δ mec1Δ*	2.9	208
3799	*rad17Δ tel1Δ*	<0.1	144
3821	*rad17Δ mec1Δ*	41.8	208
SBY#^2^			
3870	*XRS2-13myc*	99.3	140
3930	*XRS2-13myc pch2Δ*	94.5	200
4235	*XRS2-13myc rad17Δ*	26.5	204
4270	*xrs2ΔN-13myc*	89.7	204
4276	*xrs2ΔN-13myc pch2Δ*	74.5	216
4273	*xrs2ΔN-13myc rad17Δ*	1.4	216
3763	*spo11/spo11yf*	68.3	700
3866	*spo11/spo11yf tel1Δ*	50.1	688
4063	*spo11/spo11yf pch2Δ*	35.8	212
4155	*spo11/spo11yf pch2Δ tel1Δ*	3.8	208
3572	*spo11-HA*	96.0	400
3862	*spo11-HA tel1Δ*	85.3	340
4059	*spo11-HA pch2Δ*	77.5	204
4151	*spo11-HA pch2Δ tel1Δ*	46.6	204

^1^MATa/MATα ho::hisG/″ leu2::hisG/″ ura3(ΔSma-Pst)/ ″ his4-X::LEU2-(NBam)-URA3/HIS4::LEU2-(NBam); all mec1Δ strains also contain sml1Δ

2 MATa/MATα ho::hisG/″ lys2/″ leu2::hisG/″ ura3Δ::hisG/″ trp1::hisG/″ GAL3/″; spo11/spo11yf  =  spo11-HA/spo11Y135F-HA

3 Four-spore tetrads were analyzed.

**Figure 3:**
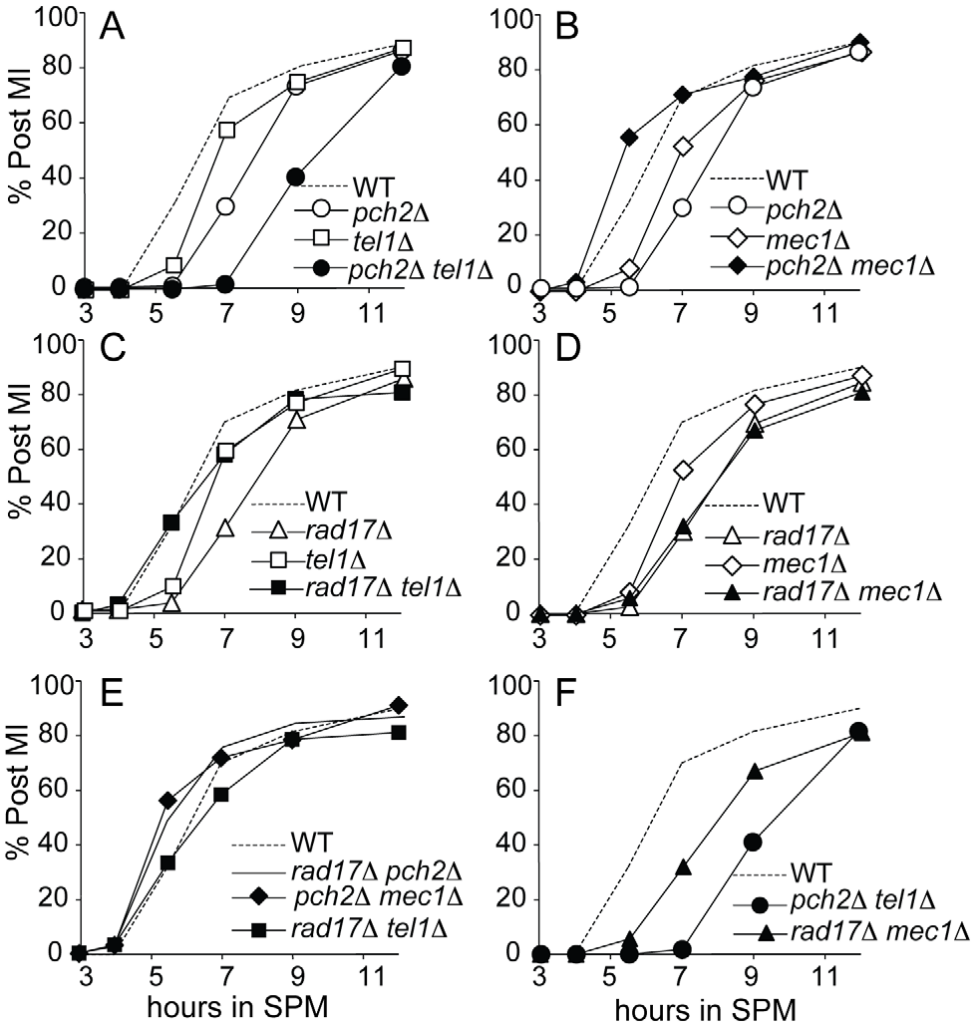
Meiotic progression analysis in single and double mutant strains. (A–F) Percentage of cells that have completed at least the first meiotic division (post-MI) in indicated strains. All data were from the same time course experiment.

In a similar line of reasoning, checkpoint activation leads to a delay in MI division and can be triggered by loss of either Pch2 or Rad17, but not both. We showed previously that MI division kinetics in the *pch2Δ rad17*Δ double mutant is faster than in wild type, yet is delayed in the two single mutant strains [20]. From a first approximation, the epistasis pattern described above for spore inviability holds true: i) the MI delay conferred by *pch2*Δ was suppressed by *mec1*Δ to give divisions even faster than WT; and ii) the delay phenotype conferred by *rad17*Δ was suppressed by *tel1*Δ (Figure 3C and 3D). Notably, the MI delay in the *pch2Δ tel1Δ* double mutant was more severe than either single mutant, suggesting that each protein may function in additional pathways that do not involve the other.

**Figure 4:**
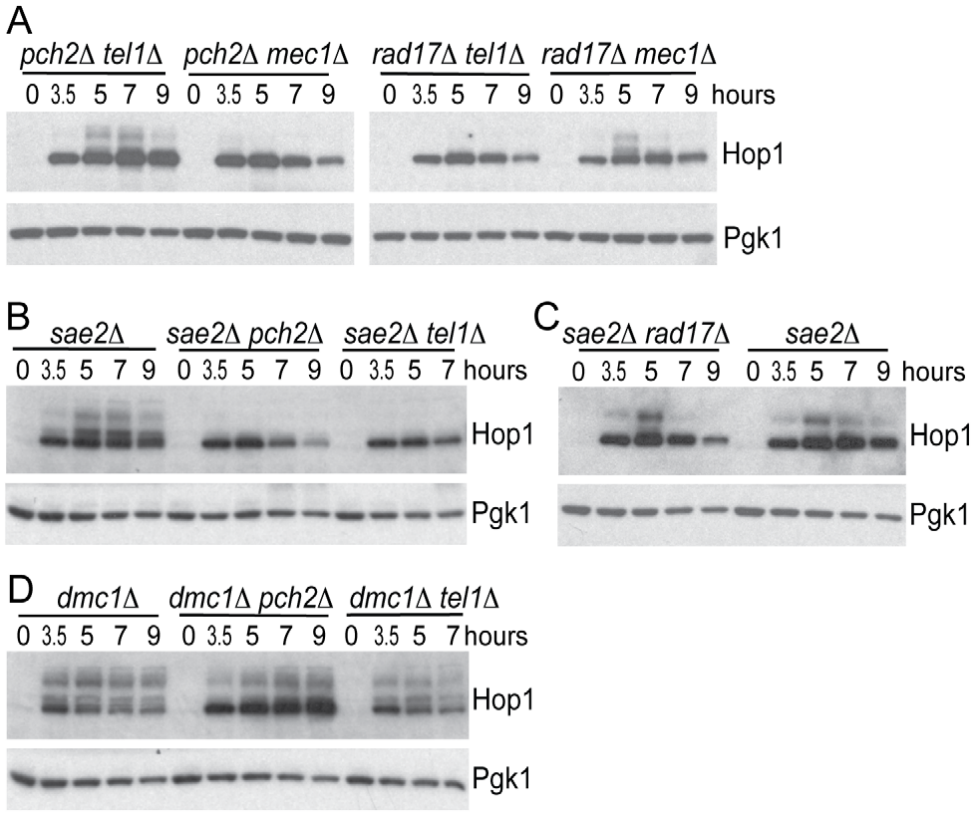
Hop1 phosphorylation in various mutants. (A–D) Hop1 phosphorylation was analyzed in indicated strains using α-Hop1 antibody similar to Figure 2A.

To further confirm the epistasis relationship observed above, we examined Hop1 phosphorylation in the *pch2Δ tel1Δ*, *rad17Δ mec1Δ*, *pch2Δ mec1Δ* and *rad17Δ tel1Δ* double mutant combinations. As expected, we observed abundant Hop1 phosphorylation in *pch2Δ tel1Δ* and *rad17Δ mec1Δ*, while only a low level of Hop1 phosphorylation was seen in *pch2Δ mec1Δ* and *rad17Δ tel1Δ* which showed very low spore viability and fast meiotic progression (Figure 3E, 3F and Figure 4A). Together, these results suggest Pch2 acts together with Tel1 to promote an essential meiotic process, perhaps by ensuring interhomolog bias through Hop1 phosphorylation.

### Pch2 is involved in signaling unprocessed DSBs

Tel1 is required to signal the presence of unprocessed DSBs during meiosis [17], [18]. Specifically, deletion of *TEL1* eliminates the signaling of unresected DSBs to Hop1 [12]. To test if signaling of unprocessed DSBs also requires Pch2, we examined Hop1 phosphorylation in both *pch2Δ* and *tel1Δ* mutants in a *sae2Δ* mutant background where breaks are unprocessed to give blunt ends. Hop1 was phosphorylated in a *sae2*Δ mutant but not in *sae2Δ pch2Δ* or *sae2Δ tel1Δ* (Figure 4B), as expected if Tel1 and Pch2 are specifically required for unprocessed DSBs signaling. By contrast, Rad17 was not required for Hop1 phosphorylation in the *sae2Δ* background (Figure 4C), which is also expected since Rad17 is involved in signaling resected DSBs. As a control, we measured Hop1 phosphorylation in the *dmc1*Δ mutant background where DSBs are resected to give ssDNA. Hop1 phosphorylation was not affected in *dmc1Δ pch2*Δ and *dmc1Δ tel1Δ* (Figure 4D).

We noticed that Hop1 protein levels were elevated in the *dmc1Δ pch2Δ* double mutant (Figure 4D) compared to the *dmc1Δ* single mutant. On the other hand, *sae2Δ pch2Δ* showed no increase in Hop1 levels compared to the *sae2Δ* single mutant (Figure 4B). We reasoned that this effect of *pch2Δ* does not relate to the role of Pch2 in promoting Hop1 phosphorylation *per se* since *pch2Δ* only affected Hop1 phosphorylation in the *sae2Δ* background (where Hop1 levels were not altered) but not in the *dmc1Δ* background (where Hop1 levels were increased). The *tel1Δ* strain did not show such an effect either, again suggesting this aspect of Pch2 function is independent of its role in Tel1 signaling to Hop1. We speculate that the increase in Hop1 levels (or reduced Hop1 protein turnover) shown here by western blotting likely reflects altered Hop1 abundance/distribution shown previously by immunostaining [21] and is related to Pch2′s role in axis organization and CO control. Interestingly, this effect is manifested at a “post resection” stage of DSB repair since increased Hop1 levels were observed in *dmc1Δ* but not in *sae2Δ*. CO designation is also thought to occur around this stage of meiotic prophase [38], [39].

### Pch2 physically interacts with the region of Xrs2 containing putative BRCT repeats

**Figure 5:**
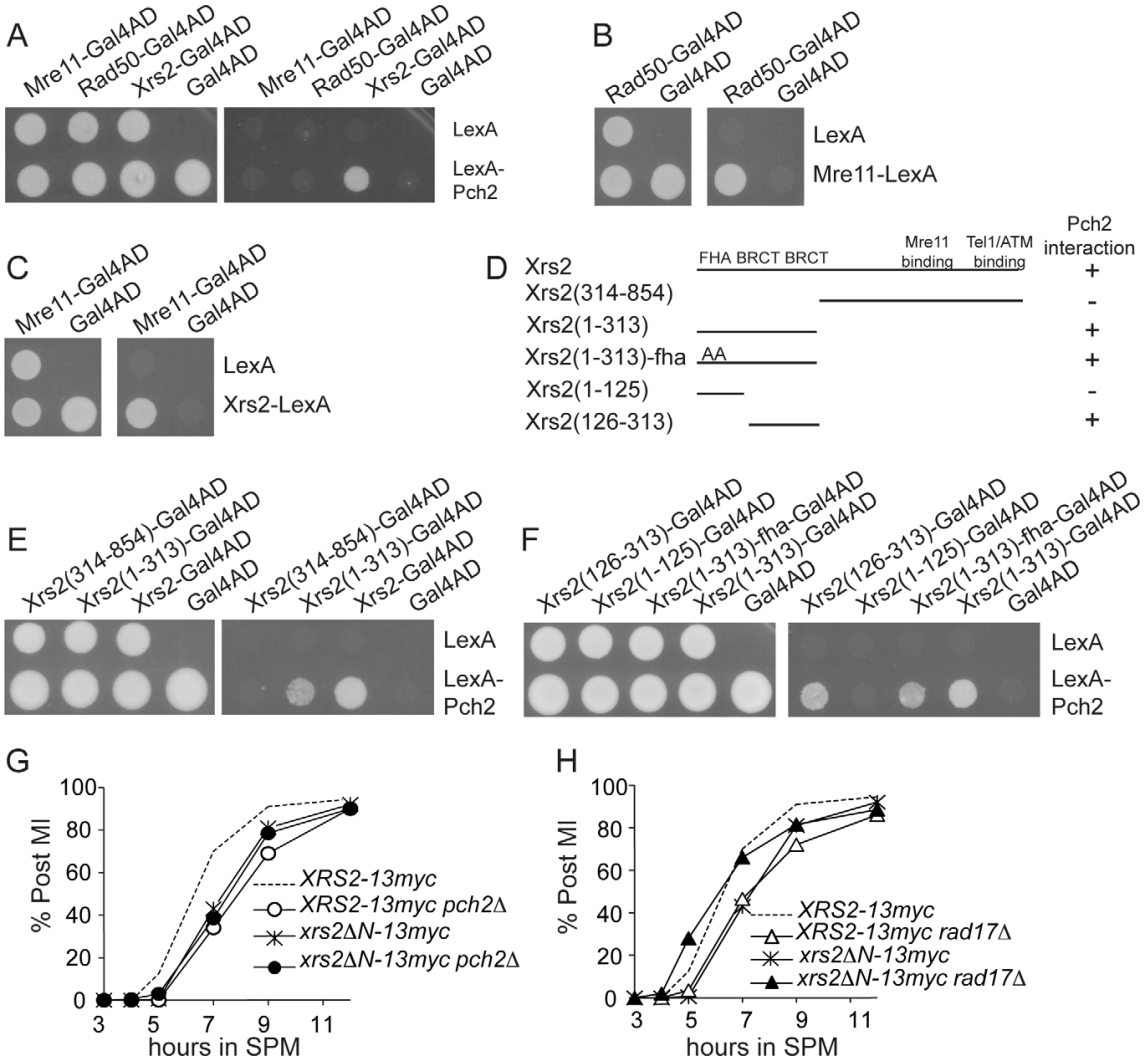
Pch2 interacts with putative BRCT repeats of Xrs2. (A–C) Two-hybrid spot assay of Pch2 and MRX complex components. Transformants carrying both LexA DNA binding domain (LexA)- and Gal4 activation domain (Gal4AD)-fusions were spotted on SC-Leu-Trp (left) and SC-Leu-Trp-His plus 1mM 3-AT (right) plates. (D–F) Mapping of the Pch2-interacting region of Xrs2. Xrs2(1–313)-fha: Two amino acids (S47 and T50) in the FHA domain were altered to alanine, shown as “AA” in (D). (G–H) Percentage of cells that have completed at least the first meiotic division (post-MI) in indicated strains. *xrs2ΔN-13myc* encodes 13myc-tagged Xrs2(314–854). Data were from the same time course experiment.

The ATM homolog Tel1 physically interacts with Xrs2 and promotes the phosphorylation of Sae2 and Hop1 [12], [40], [41]. We thus tested if Pch2 also interacts with components of the MRX complex using pair-wise bait-prey combinations of Pch2 with Mre11, Rad50 and Xrs2 for yeast two-hybrid analysis. In this trial, Pch2 interacted with Xrs2, but not Mre11 or Rad50 (Figure 5A). Mre11 and Rad50 two-hybrid constructs were functional since we detected interaction between Rad50-Mre11 (Figure 5B) and Mre11-Xrs2 (Figure 5C). We narrowed the Pch2-binding region of Xrs2 to a 187 amino acid region in the Xrs2(126–313)-Gal4AD construct (Figure 5D – 5F). This region contains two putative BRCT repeats, similar to the human ortholog Nbs1 [42]. Point mutations created to abolish FHA domain function present in Xrs2(1–313)-Gal4AD did not abolish interaction with LexA-Pch2 [43] (Figure 5F).

### *xrs2*Δ*N* recapitulates *pch2*Δ effects on Hop1 phosphorylation and spore viability

The first 313 amino acids of Xrs2 are dispensable for the formation of normal levels of DSBs and crossover recombination products yet DSB turnover and MI division are delayed [44]. We created the allele *xrs2ΔN-13myc* that deleted the first 313 amino acid coding region of *XRS2* and found it delayed MI division (Figure 5G and 5H), presumably due to the slow turnover of DSBs as in the *pch2*Δ mutant [20], [21], [45]. We wondered if *xrs2ΔN-13myc,* like *pch2Δ,* would suppress the MI delay conferred by *rad17*Δ (and vice versa). We found this to be the case with MI division timing in *xrs2ΔN-13myc rad17*Δ occurring earlier than either single mutant (Figure 5H). By contrast, MI division was delayed in *xrs2ΔN-13myc pch2*Δ (Figure 5G).

Spore viability of *xrs2ΔN-13myc rad17*Δ (1.4%; Table 1) was dramatically decreased compared to *xrs2ΔN-13myc* and *XRS2-13myc rad17*Δ (89.7% and 26.5%, respectively), while *xrs2ΔN-13myc pch2*Δ gave only a modest reduction of spore viability compared to *XRS2-13myc pch2*Δ (74.5% and 94.5%, respectively).

**Figure 6:**
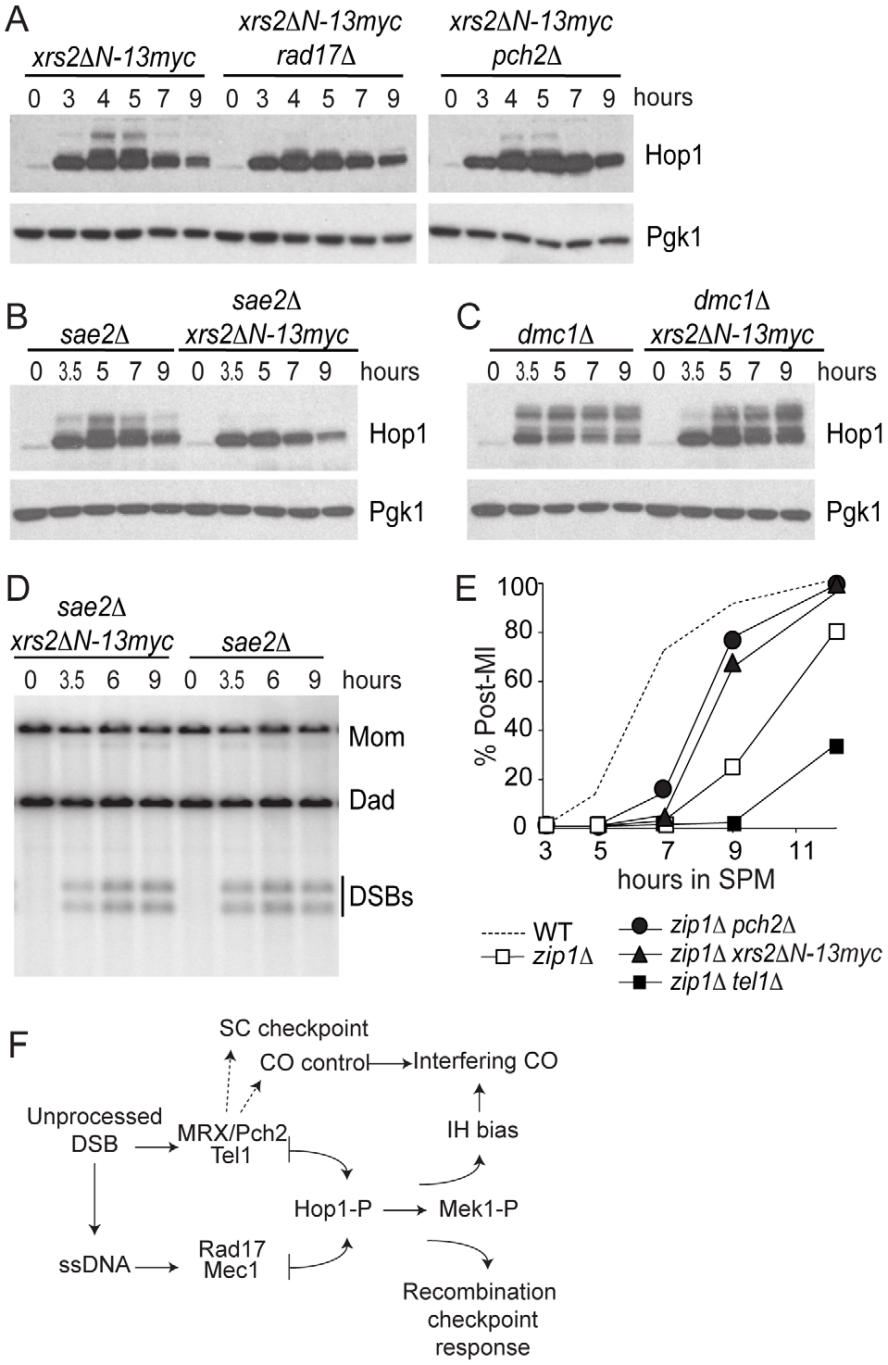
*xrs2ΔN-13myc* phenocopies *pch2Δ.* (A–C) Hop1 phosphorylation was analyzed in indicated strains using α-Hop1 antibody similar to Figure 2A. (D) Southern blot of 1D gel analysis of DSBs in *sae2Δ* and *sae2Δ xrs2ΔN-13myc*. (E) Percentage of cells that have completed at least the first meiotic division (post-MI) in indicated strains. (F) Model; see text for details.

To test if *xrs2ΔN-13myc* affects checkpoint signaling in a similar manner to *pch2Δ*, we examined the effect of this mutation on Hop1 phosphorylation in *xrs2ΔN-13myc rad17Δ* and *xrs2ΔN-13myc pch2*Δ double mutants as well as in *sae2Δ* and *dmc1Δ* backgrounds. We found Hop1 phosphorylation was greatly reduced in *xrs2ΔN-13myc rad17Δ* but not in *xrs2ΔN-13myc pch2*Δ (Figure 6A). Furthermore, *xrs2ΔN-13myc* only abrogated Hop1 phosphorylation in *sae2Δ* but not in *dmc1Δ* backgrounds (Figure 6B and 6C). The absence of Hop1 phosphorylation in *sae2Δ xrs2ΔN-13myc* was not due to reduced DSB levels (Figure 6D). Notably, as with *dmc1Δ pch2Δ*, *dmc1Δ xrs2ΔN-13myc* accumulated more Hop1 protein (Figure 6C). Taken together, these results suggest that the interaction of Pch2 with the N-terminal region of Xrs2, and perhaps the putative BRCT repeats specifically, is required for Pch2′s role(s) in the recombination checkpoint and axis organization during meiosis.

### *xrs2ΔN* but not *tel1Δ* recapitulates *pch2*Δ effects on the *zip1Δ*-induced MI delay

Budding yeast *pch2*Δ was originally isolated in the BR background as a mutation that suppresses the meiotic arrest that occurs in the absence of Zip1 [31] and Pch2 has been thought to be involved in a “synapsis checkpoint” [46]. In SK1, *zip1Δ* caused a meiosis I delay that is partially suppressed by *pch2Δ* [20]. We found that *xrs2ΔN-13myc,* but not *tel1Δ*, suppressed the *zip1Δ*-induced meiotic delay, suggesting that interaction with Xrs2 may also be required for Pch2′s role in the synapsis checkpoint (Figure 6E). In fact MI delays conferred by *tel1Δ* and *zip1Δ* are independent since the double mutant exhibits a more severe delay. It is thus possible that Xrs2-Pch2 interaction is required for most, if not all, functions of Pch2; while Pch2 and Tel1 may perform independent functions besides their concerted role in the recombination checkpoint.

### Pch2 and Tel1 regulate spore viability independently when DSBs are reduced

Deletion of *PCH2* has been shown to sensitize strains carrying hypomorphic alleles of *spo11* to give lower levels of spore viability [22], . Through our studies we found that the deletion of *TEL1* also gave a modest reduction of spore viability in a *spo11-HA/spo11Y135F-HA* background (50.1% versus 68.3%; Table 1), but not to the extent of *pch2*Δ (35.8%). When both Pch2 and Tel1 are absent, spore viability was dramatically reduced in this background (3.8%). Similar effects were also observed in the *spo11-HA* homozygous mutant background (Table 1). These data suggest that Pch2 and Tel1 independently influence an essential meiotic process that is sensitive to DSB levels. Identification of this process will require analysis of the *pch2Δ tel1Δ spo11-HA/spo11Y135F-HA* strain for defects in other meiotic chromosome events including DSB repair, crossover control, chromosome axis morphogenesis and/or synapsis.

## Discussion

This work presents evidence defining new functions for Pch2 and Xrs2 in promoting proper chromosome segregation during meiosis: First, Pch2 and Xrs2 function in the same epistasis pathway as Tel1 (ATM) to activate the recombination checkpoint in response to unprocessed DSBs. Second, Pch2 and Xrs2 function together in the synapsis checkpoint, independent of Tel1. Third, Pch2 interacts with the N-terminus of Xrs2 containing tandem BRCT repeats. The N-terminus of Xrs2 is required to activate both the recombination and synapsis checkpoints. Finally, a role for Pch2 in preventing intersister recombination events is revealed when one branch of the recombination checkpoint is abolished by deletion of *RAD17*. The separate roles for Pch2 and Rad17 in mediating the recombination checkpoint via sequential phosphorylation of Hop1 and Mek1 may account for their combined role in preventing intersister repair of DSBs. These findings may help to address the seemingly disparate roles Pch2 plays among synaptic organisms, including meiotic recombination, chromosome axis formation, checkpoint signaling and crossover control [21], [47].

### Recombination checkpoint

We propose that phosphorylation of the meiotic chromosome axis protein Hop1 is regulated by two partially redundant pathways: one pathway requires Tel1, Pch2 and Xrs2 and responds to the presence of unprocessed DSBs; a second pathway requires Mec1 and Rad17 and responds to the presence of resected DSB intermediates of homologous recombination (Figure 6F). This model is directly analogous to the different roles of Tel1/Xrs2 and Mec1/Rad17 in the DNA damage response during vegetative growth [18] with Pch2 providing a regulatory feature specific to meiotic chromosomes that coordinate the events of meiotic recombination with axis organization.

The physical association of Pch2 with Xrs2 suggests a mechanism to promote interhomolog bias near sites of DSBs by bringing the Tel1/ATM kinase near its substrate Hop1, a component of the chromosome axis. Pch2 might utilize the binding and/or hydrolysis of ATP to promote conformational changes in axis structure that enable the phosphorylation of Hop1 by Tel1. Alternatively or in addition, Pch2 interaction with Xrs2 might function to stabilize the association of the MRX complex at the chromosome axis analogous to the interaction of Mdc1 protein (Mediator of DNA damage Checkpoint) with the BRCT repeats of the mammalian Xrs2 ortholog, Nbs1 [48]. In this case, Mdc1 stabilizes the association of Nbs1 at sites of DNA damage, thus creating a microenvironment to promote phosphorylation of H2AX by ATM [49].

It is not clear if Pch2 interacts with Xrs2 (Nbs1) in other organisms. The mouse ortholog of Pch2, TRIP13, is implicated in early recombination steps that follow DSB resection but precede Rad51 focus formation [25]. It is possible that TRIP13-NBS1 interaction could establish a precondition that facilitates a later step of recombination. Indeed in yeast, deletion of *PCH2* results in the slow turnover of resected DSBs [20], [21], [45]. In *Drosophila*, NBS is required for DSB repair [50], but its role in meiotic recombination has not been explored to date. While Xrs2/Nbs1 proteins are conserved from vertebrates to fungi, there is no apparent ortholog in *C. elegans*. It remains possible that Pch2 plays a role in a recombination checkpoint in *C. elegans* that has not yet been uncovered experimentally.

### Synapsis checkpoint

Pch2 orthologs in worm and fly are implicated in a checkpoint activated by the failure to synapse chromosome and/or by disruptions in axis formation. The synapsis checkpoint functions in these instances even in absence of DSB formation [28], [30]. Although synapsis is dependent on DSB formation in budding yeast, several examples implicate Pch2 in a synapsis checkpoint that responds to defects in synapsis and/or axis structure in situations where DSBs are efficiently repaired [13], [51]. Strong evidence in support of a synapsis checkpoint comes from our previous observation that *MEK1-GST,* an artificially activated form of *MEK1*, acts as a genetic enhancer of *zip1Δ* by causing MI arrest [13]. Since DSBs are efficiently repaired in this situation [13], this result suggests that synapsis and/or axis defects trigger the arrest, not the persistence of unrepaired DNA breaks. Deletion of *PCH2*, but not *TEL1*, can bypass this arrest, suggesting an independent role for Pch2 in a synapsis checkpoint (unpublished data). Similarly, deletion of *PCH2* suppresses the meiosis I arrest phenotype that is activated by the presence of aberrantly synapsed chromosomes caused by the non-null allele *zip1-4LA,* which also repairs DNA breaks efficiently [51]. We found here that *xrs2ΔN-13myc*, similar to *pch2Δ*, partially suppressed *zip1Δ* delay, suggesting that Xrs2, perhaps through association with Pch2, is required to execute the synapsis checkpoint. By contrast, Tel1 does not seem to be involved in this branch of Pch2′s function. Borner and colleagues argued previously that Pch2 might mediate Mec1/ATR activity with respect to sensing “structure-dependent interchromosome interactions” [21]. It is possible that a Pch2/Xrs2/Mec1 pathway functions in this program.

Further understanding of the differential requirements for Xrs2 and Tel1 for Pch2 function in the recombination checkpoint versus the synapsis checkpoint (and possibly crossover control) may help to identify common mechanisms shared among synaptic organisms, where pairing and SC formation are not always coupled to recombination [52].

## Materials and Methods

### Strains

All strains are derivatives of SK1 except the strain used for yeast two hybrid spot assay is L40 (*MATa trp1 leu2 his3 LYS2::lexA-HIS3 URA3::lexA-lacZ*) [53]. Deletion mutants were generated by PCR-based gene disruption [54], [55]. All the *mec1Δ* strains also carried *sml1Δ* to suppress inviability. *MEK1-3HA* and *XRS2-13myc* were made by using pFA6a-3HA-kanMX6 and pFA6a-13myc-kanMX6 modules, respectively [56]. *xrs2ΔN-13myc* was created by two-step allele replacement. Briefly, a PCR-amplified *URA3* was used to replace the region encoding amino acid 1–313 of Xrs2-13myc. Then a PCR-generated fragment containing 385 bp upstream the first coding ATG fused to 375 bp downstream the ATG encoding amino acid 314 of Xrs2 was used to replace the *URA3*, resulting in *xrs2ΔN-13myc* expressing 13myc-tagged Xrs2(314–854) under the native promoter of *XRS2*. *spo11-HA* and *spo11Y135F-HA* are a gift from Scott Keeney and crossed into our strain background.

SBY strain numbers are listed in Table 1. Additional strains used in this study are: strains isogenic to SBY1903 (*MATa/MATα ho::hisG/*″ *leu2::hisG/*″ *ura3(ΔSma-Pst)/*″ *his4-X::LEU2-(NBam)-URA3/HIS4::LEU2-(NBam)*) except the indicated mutations: SBY3055 (*ntd80Δ*); SBY3280 (*ntd80Δ pch2Δ*); SBY3277 (*ntd80Δ rad17Δ*); SBY3274 (*ntd80Δ pch2Δ rad17Δ*); SBY2591 (*dmc1Δ*); SBY2597 (*dmc1Δ pch2Δ*); SBY2594 (*dmc1Δ rad17Δ*); SBY2606 (*dmc1Δ pch2Δ rad17Δ*); SBY3800 (*dmc1Δ tel1Δ*); SBY2611 (*sae2Δ*); SBY2625 (*sae2Δ pch2Δ*); SBY3843 (*sae2Δ tel1Δ*); SBY2616 (*sae2Δ rad17Δ*); SBY4684 (*sae2Δ xrs2ΔN-13myc*); SBY3589 (*MEK1-3HA*); SBY3595 (*MEK1-3HA pch2Δ*); SBY3592 (*MEK1-3HA rad17Δ*); SBY3598 (*MEK1-3HA pch2Δ rad17Δ*); strains isogenic to SBY4056 (*MATa/MATα ho::hisG/*″ *lys2/*″*leu2::hisG/*″ *ura3Δ::hisG/*″ *trp1::hisG/*″ *GAL3/*″) except the indicated mutations: SBY3560 (*dmc1Δ*), SBY4517 (*dmc1Δ xrs2ΔN-13myc*), SBY3644 (*sae2Δ*), SBY4514 (*sae2Δ xrs2ΔN-13myc*), SBY4445 (*zip1Δ*), SBY4451 (*zip1Δ pch2Δ*), SBY4448 (*zip1Δ tel1Δ*), SBY4404 (*zip1Δ xrs2ΔN-13myc*); *tel1Δ*, *sml1Δ*, and *ndt80Δ* are marked with *hphMX*; *meclΔ* is marked with *natMX*; all other mutations are marked with *kanMX*.

### Sporulation conditions

Time courses were conducted by the SPS method [20]. Briefly, cells were patched on YPG plates (3% glycerol, 2% bactopeptone, 1% yeast extract, 2% bactoagar, 0.01% adenine sulphate, 0.004% tryptophan) for ∼14 hr and then stripped on YPD plates (2% glucose, 2% bactopeptone, 1% yeast extract, 2% bactoagar, 0.01% adenine sulphate, 0.004% tryptophan) and grown for 2 days. Single colonies were used to inoculate 5 ml YPD (plus 0.002% uracil if *ura3* strains were used) liquid cultures and grown for at least 24 hr before diluted into SPS (1% potassium acetate, 0.5% yeast extract, 1% bactopeptone, 0.17% yeast nitrogen base, 0.5% ammonium sulphate, 1.02% potassium biphthalate) (plus 0.002% uracil if *ura3* strains were used) at O.D.600 = 0.16. SPS cultures were grown for ∼15.5 hr, washed with H_2_O, and then resuspended into SPM (1% potassium acetate, 0.02% raffinose, 0.009% amino acid powder) at O.D.600 = 2–3. Spore viability data were obtained by sporulation on solid SPM media. All procedures were performed at 30°C.

### DNA physical assay and meiotic progression analysis

DNA extraction, gel electrophoresis and southern blot were performed as previously described [9]. Meiosis I division timing was determined by calculating the percentage of post-MI cells at indicated time points. Briefly, meiotic cultures were fixed in 50% ethanol and stained with DAPI. Cells with more than 2 DAPI-stained nucleus bodies were counted as post-MI cells. 200 cells were counted for each time points.

### Protein extraction, Western blotting, and immunoprecipitation

Denaturing whole-cell extracts were prepared as previously described [57] with modifications. Briefly, 1 mL meiotic cultures at indicated time points were spun down and resuspended in 1 mL ice-cold water with 1 mM PMSF, 10 mM sodium fluoride and 10 mM sodium diphosphate. 150 µL ice-cold 2 N NaOH / 8% 2-ME was then added and mixtures were incubated on ice for 10 min. After added 160 µL ice-cold 50% TCA and incubated on ice for 10 min, mixtures were spun for 8 min at 14000 rpm. Pellets were washed by 500 µL ice-cold acetone and spun for 5 min at 14000 rpm. Washed pellets were dried by spinning in the vacufuge for 8 min and then resuspended in 1X SDS sample buffer with PMSF, sodium fluoride and sodium diphosphate. A bath sonicator was used to facilitate resuspension in acetone and sample buffer. Proteins from denaturing whole-cell extracts were detected by Western blotting using α-Hop1 (S. Roeder), α-HA (Santa Cruz, sc-7392), α-phospho-Akt substrate (Cell signaling, #9614), and α-Pgk1 (Invitrogen, A-6457). Immunoprecipitation was performed as previously described [13] except α-HA antibody (Santa Cruz, sc-7392) was used.

### Plasmids and yeast two hybrid analysis

Mre11, Rad50, and Xrs2 yeast two-hybrid plasmids are a gift from S. Keeney [58]. Xrs2 truncation plasmids were constructed by cloning PCR-generating fragments into the same plasmid for full-length Xrs2 (pACT2-2). Xrs2(1–313)-S47A H50A plasmid was created by QuikChange (Stratagene). LexA-Pch2 plasmid was made by cloning PCR amplified intronless *PCH2* coding region into pCA1 plasmid. Y2H spot assay was performed by spotting 5 µL O.D._600_  = 1 cultures onto SC-Leu-Trp plates and SC-Leu-Trp-His + 1mM 3AT plates and grown for 3–5 days.
